# Lung function in the absence of respiratory symptoms in overweight children and adolescents*

**DOI:** 10.1590/S1806-37132014000200006

**Published:** 2014

**Authors:** Silvana Neves Ferraz de Assunção, Carla Hilário da Cunha Daltro, Ney Christian Boa Sorte, Hugo da Costa Ribeiro, Maria de Lourdes Bastos, Cleriston Farias Queiroz, Antônio Carlos Moreira Lemos

**Affiliations:** Professor Edgard Santos University Hospital, Federal University of Bahia School of Medicine, Salvador, Brazil; Professor Edgard Santos University Hospital, Federal University of Bahia School of Medicine, Salvador, Brazil; Professor Edgard Santos University Hospital, Federal University of Bahia School of Medicine, Salvador, Brazil; Professor Edgard Santos University Hospital, Federal University of Bahia School of Medicine, Salvador, Brazil; Professor Edgard Santos University Hospital, Federal University of Bahia School of Medicine, Salvador, Brazil; Professor Edgard Santos University Hospital, Federal University of Bahia School of Medicine, Salvador, Brazil; Professor Edgard Santos University Hospital, Federal University of Bahia School of Medicine, Salvador, Brazil

**Keywords:** Obesity, Respiratory function tests, Lung diseases

## Abstract

**OBJECTIVE:**

To describe lung function findings in overweight children and adolescents without respiratory disease.

**METHODS:**

This was a cross-sectional study involving male and female overweight children and adolescents in the 8-18 year age bracket, without respiratory disease. All of the participants underwent anthropometric assessment, chest X-ray, pulse oximetry, spirometry, and lung volume measurements. Individuals with respiratory disease were excluded, as were those who were smokers, those with abnormal chest X-rays, and those with an SpO_2_ = 92%. Waist circumference was measured in centimeters. The body mass index-for-age Z score for boys and girls was used in order to classify the individuals as overweight, obese, or severely obese. Lung function variables were expressed in percentage of the predicted value and were correlated with the anthropometric indices.

**RESULTS:**

We included 59 individuals (30 males and 29 females). The mean age was 11.7 ± 2.7 years. Lung function was normal in 21 individuals (35.6%). Of the 38 remaining individuals, 19 (32.2%), 15 (25.4%), and 4 (6.7%) presented with obstructive, restrictive, and mixed ventilatory disorder, respectively. The bronchodilator response was positive in 15 individuals (25.4%), and TLC measurements revealed that all of the individuals with reduced VC had restrictive ventilatory disorder. There were significant negative correlations between the anthropometric indices and the Tiffeneau index in the individuals with mixed ventilatory disorder.

**CONCLUSIONS:**

Lung function was abnormal in approximately 65% of the individuals evaluated here, all of whom were overweight. Obstructive ventilatory disorder and positive bronchodilator response predominated.

## Introduction

Obesity is a multifactorial chronic disease that is epidemic worldwide.^(^ ^1^ ^)^ Because of the socioeconomic impact, morbidity, and mortality of obesity, there has been growing interest in understanding the disease.^(^ ^2^ ^)^ Impaired lung function is a possible complication of obesity and is often overlooked, despite occurring in a manner that is similar to that observed in other diseases, such as cancer, cardiovascular disease, and chronic respiratory disease.^(^ ^3^ ^)^ Lin at al.^(^ ^4^ ^)^ reported that various obesity-related cytokines can contribute to systemic inflammatory effects in individuals with obstructive airway disease and sleep apnea syndrome.

Although obesity-related lung function changes have been described in adults, there is a lack of data on such changes in children.^(^ ^5^ ^)^ The increase in childhood obesity is an emerging problem worldwide and directly contributes to obesity in adulthood; as a result, there is an increase in the incidence of fatal diseases such as cardiovascular disease, metabolic syndrome, dyslipidemia, diabetes mellitus, arterial hypertension, and even respiratory changes.^(^ ^6^ ^)^

Methodological differences across studies in terms of the diagnostic evaluation of obesity and the selection of a well-characterized population make direct comparisons difficult and underscore the need for further studies. In view of these considerations, the objective of the present study was to describe lung function changes in overweight children and adolescents without respiratory symptoms.

## Methods

This was a cross-sectional descriptive study involving male and female overweight patients in the 8-18 year age bracket. Age was recorded in completed whole years, and skin color/ethnicity was classified as black (African), brown (Mulatto), white (Caucasian), yellow (Asian), or red (Indigenous) on the basis of the parameters established by the Brazilian Institute of Geography and Statistics.^(^ ^7^ ^)^ A convenience sample was recruited between May of 2010 and September of 2011 from among patients treated at the child and adolescent obesity outpatient clinic of the Pediatric Clinical Nutrition Department of the Professor Edgard Santos University Hospital, located in the city of Salvador, Brazil. The participants completed a systematic questionnaire designed for the present study and including data obtained during history taking and physical examination (with emphasis on respiratory findings), ancillary tests being subsequently performed. The inclusion criteria were as follows: having exogenous obesity; having no respiratory symptoms; having normal pulmonary auscultation and chest X-ray findings; and having a baseline SpO_2_ > 92 %. Neither smokers nor patients with a history of wheezing, cough, chest pain, or respiratory disease were included in the present study.

Waist circumference was measured with a flexible, nonelastic tape measure at the midpoint between the lower costal margin and the anterior superior iliac crest.^(^ ^8^ ^)^ Two measurements were performed, and the mean was used. Participants were weighed (in their underwear) on a portable scale (Filizola, São Paulo, Brazil) accurate to within 100 g. Height (in cm) was measured to the nearest 0.1 cm with a stadiometer with a base plate. The body mass index (BMI) was calculated by the formula weight/height^2^ (kg/m^2)^, and the World Health Organization (WHO) BMI-for-age Z score for girls and boys^(^ ^9^ ^)^ was used to classify the participants as overweight (having a BMI > 1 SD above the WHO growth standard median), obese (having a BMI > 2 SDs above the WHO growth standard median), or severely obese (having a BMI > 3 SDs above the WHO growth standard median).

Lung function was assessed in the Pulmonary Function Laboratory of the Federal University of Bahia, located in the city of Salvador, Brazil. Lung function was assessed by spirometry with pharmacological testing, spirometry being performed with a Koko Digidoser pneumotachograph spirometer (Ferraris Respiratory, Louisville, CO, USA). The tests were performed before and 15 min after the administration of 400 µg of inhaled albuterol. The helium dilution method was used in order to measure RV and TLC, which were measured with a Vmax 21 mass flow sensor (Viasys Healthcare, Palm Springs, CA, USA). Participants were advised to refrain from coffee, tea, medications (such as bronchodilators), and large amounts of food before the tests. Participants performed the tests with their heads in a central position, using a nose clip. All tests were performed at the same time of day (in the afternoon) by the same professional. At least three FVC maneuvers were performed by each individual; the tests and curves that met the acceptability and reproducibility criteria of the American Thoracic Society^(^ ^10^ ^)^ and the Brazilian Thoracic Association^(^ ^11^ ^)^ were selected. The highest of at least three technically acceptable measurements of FEV_1_ and FVC were selected. Those were expressed as absolute values and as percentages of predicted values, the latter being defined as the lower limit of normal (LLN) for each individual. The LLN was calculated by the equation described by Pereira,^(^ ^12^ ^)^ valid for both genders. The reference values for lung volumes (RV and TLC) were those reported by Pennock et al.^(^ ^13^ ^)^

 On the basis of spirometric values, TLC, and RV, patients were classified as having one of the following:

- normal lung function-FVC = LLN; FEV_1_/FVC = LLN; and normal RV and TLC
- obstructive lung disease (OLD)-FEV_1_/FVC < LLN; normal or increased RV or TLC; or a positive bronchodilator response
- restrictive lung disease (RLD)-FVC < LLN; FEV_1_/FVC = LLN; and reduced RV and TLC
- mixed obstructive and restrictive lung disease (MORLD)-FEV_1_/FVC < LLN and reduced TLC

Bronchodilator response testing was performed in accordance with the Brazilian Thoracic Association guidelines,^(^ ^11^ ^)^ a significant bronchodilator response being characterized by the following:

- abnormal spirometry results showing a post-bronchodilator absolute change in FEV_1_ (post-bronchodilator FEV_1_ - pre-bronchodilator FEV_1_) = 200 mL; an absolute variation in FEV_1_ in relation to the predicted value ([post-bronchodilator FEV_1_ - pre-bronchodilator FEV_1_] × 100/predicted FEV_1_) > 7%; and a post-bronchodilator absolute change in FVC (post-bronchodilator FVC - pre-bronchodilator FVC) = 350 mL
- normal spirometry results showing a post-bronchodilator change in FEV_1_ (post-bronchodilator FEV_1_ - pre-bronchodilator FEV_1_) = 10%

Data analysis was performed with the IBM SPSS Statistics software package, version 19.0 (IBM Corporation, Armonk, NY, USA). Quantitative variables were expressed as mean ± standard deviation. Qualitative variables were expressed as absolute and relative frequencies. Pearson's test was used in order to assess correlations among spirometric variables, BMI-for-age Z scores, and waist circumference measurements in the OLD, RLD, and MORLD groups. Values of p = 0.05 were considered significant. The present study was approved by the local research ethics committee (Ruling no. 80/09), and the parents or legal guardians of all participants gave written informed consent.

## Results

The study sample consisted of 59 individuals, the mean age being 11.7 ± 2.7 years. Of those 59 individuals, 30 (50.8%) were male. In addition, 32 (54.2%) were Mulatto, 22 (37.2%) were Black, and 5 (8.4%) were White. The BMI-for-age Z scores ranged from 1.2 SDs to 6.1 SDs, with a mean of 3.1 ± 1.0 (kg/m^2)^. Therefore, of the 59 individuals studied, 4 (6.7%) were classified as overweight, 28 (47.4%) were classified as obese, and 27 (45.7%) were classified as severely obese. Table 1 shows the lung function measurements in % of predicted.

**Table 1:** Lung function measurements (in percentage of predicted values) in 59 overweight children and adolescents with normal lung function, obstructive lung disease, restrictive lung disease, or mixed obstructive and restrictive lung disease.a

Lung function	FEV_1_		FVC		FEV_1_/FVC		FEF_25-75%_		TLC	RV
	Pre-Bd	Post-Bd	Pre-Bd	Post-Bd	Pre-Bd	Post-Bd	Pre-Bd	Post-Bd	Pre-Bd	Pre-Bd
Normal	95.43 ± 10.89	95.10 ± 12.19	93.05 ± 9.51	90.16 ± 11.40	86.81 ± 3.44	88.67 ± 2.79	64.86 ± 14.38	66.86 ± 15.14	91.40 ± 8.70	89.65 ± 27.43
OLD	86.26 ± 13.23	89.74 ± 17.19	93.06 ± 13.51	92.95 ± 15.50	76.42 ± 5.09	80.47 ± 7.40	51.42 ± 14.35	60.63 ± 22.30	99.00 ± 13.55	118.56 ± 47.53
RLD	75.33 ± 13.08	79.27 ± 14.54	72.20 ± 14.30	74.27 ± 15.56	88.80 ± 4.88	89.21 ± 5.80	57.33 ± 12.88	64.20 ± 18.27	69.38 ± 7.95	66.62 ± 42.53
MORLD	73.50 ± 5.91	80.50 ± 12.15	83.25 ± 4.19	86.00 ± 13.24	74.25 ± 3.86	78.75 ± 3.86	45.25 ± 8.57	55.50 ± 14.40	71.75 ± 11.89	37.25 ± 41.99

Bd : bronchodilator

OLD : obstructive lung disease

RLD : restrictive lung disease

Of the 59 individuals studied, 17 (30.3%) had TLC values < 80% of predicted, whereas 2 (3.5%) had TLC values > 120%.

Of the 59 individuals studied, 15 (25.4%) had a positive bronchodilator response (as determined by the change in FEV_1_), 10 (67.0%) of those 15 individuals being severely obese (Table 2). They all had FEV_1_/FVC values below the LLN, being therefore classified as having OLD. The change in FVC (i.e., post-bronchodilator FVC - pre-bronchodilator FVC) characterized a positive bronchodilator response in only 2 individuals, and none of the patients with MORLD had a positive bronchodilator response. One of the individuals with a positive response was unable to undergo measurement of lung volumes.

**Table 2:** Respiratory function variables (in percentage of predicted values) in 15 children and adolescents showing changes in FEV1.

Individual	FEV_1_		FVC		FEV_1_/FVC		FEF_25-75%_		TLC	RV
	Pre-Bd	Post-Bd	Pre-Bd	Post-Bd	Pre-Bd	Post-Bd	Pre-Bd	Post-Bd	Pre-Bd	Pre-Bd
01	85	94	101	102	71	78	53	65	95	62
02	84	91	87	89	79	84	58	75	86	78
03	94	102	100	101	78	83	72	80	94	71
04	101	110	108	112	80	83	70	81	104	99
05	103	110	111	114	78	82	69	82	119	150
06	100	108	101	100	81	89	80	110	89	31
07	70	94	70	95	85	83	33	40		
08	69	78	83	91	70	73	35	43	79	70
09	68	78	75	78	75	80	38	47	84	106
10	92	103	112	108	69	79	48	60	124	171
11	84	96	83	90	84	88	69	69	80	74
12	80	98	89	102	77	81	56	74	78	46
13	86	101	94	92	78	93	51	75	128	235
14	86	97	87	93	82	86	62	78	104	169
15	70	82	68	70	95	100	44	80	73	103

Bd : bronchodilator

Considering FEV_1_ a predictor of bronchial obstruction, we analyzed the changes occurring in absolute and percent predicted FEV_1_ after bronchodilator use. The results are shown in Table 3.

**Table 3:** Changes in FEV1 in the 15 overweight children and adolescents with a positive bronchodilator response.

Individual	ΔFEV_1_, mL*	ΔFEV_1_, %**
01	260	9
02	180	7
03	310	8
04	260	9
05	210	7
06	350	8
07	420	24
08	270	9
09	240	7
10	290	11
11	310	12
12	570	18
13	380	15
14	370	11
15	260	12

* Mean ± SD = 312 ± 96.7 mL

** Mean ± SD = 11.1 ± 4.7%

We examined the relationship between lung disease and the degree of obesity in the study sample. The results are shown in Table 4.

**Table 4:** Lung function in the study sample, by degree of obesity.a

Lung function	Overweight/obese	Severely obese	Total
Normal	14 (23.7)	07 (11.8)	21 (35.5)
OLD	09 (15.2)	10 (16.9)	19 (32.2)
RLD	07 (11.8)	08 (13.5)	15 (25.4)
MORLD	02 (3.3)	02 (3.3)	04 (6.7)

OLD : obstructive lung disease

RLD : restrictive lung disease

**MORLD:** : mixed obstructive and restrictive lung disease

a Values expressed as n (%)

Pearson's correlation analysis was performed in order to determine whether the anthropometric measurements (i.e., BMI and waist circumference) correlated with the spirometric variables in the OLD, RLD, and MORLD groups. The results are shown in Table 5. There was a significant negative correlation between the anthropometric measurements and the FEV_1_/FVC ratio in the individuals with MORLD.

**Table 5:** Correlations between anthropometric and spirometric variables in the obstructive lung disease, restrictive lung disease, and mixed obstructive and restrictive lung disease groups.

Spirometric variables	Anthropometric variables												
	Waist circumference							Body mass index					
	OLD		RLD		MORLD			OLD		RLD		MORLD	
	r	p	r	p	r	p		r	p	r	p	r	p
FEV_1_	0.064	0.795	0.331	0.228	0.628	0.371		-0.092	0.709	0.146	0.603	0.434	0.566
FVC	0.048	0.845	0.250	0.369	0.645	0.355		-0.127	0.603	0.091	0.747	0.461	0.539
FEV_1_/FVC	0.095	0.700	0.113	0.689	-0.996	0.004		-0.219	0.367	0.106	0.708	-0.986	0.014
FEF_25-75%_	0.055	0.823	0.265	0.341	-0.667	0.333		0.203	0.405	0.358	0.190	-0.527	0.473

OLD : obstructive lung disease

RLD : restrictive lung disease

**MORLD:** : mixed obstructive and restrictive lung disease

## Discussion

In the present study, we evaluated the lung function of overweight children and adolescents with normal chest X-rays and without respiratory symptoms or a history of respiratory disease. Pulmonary function test results were abnormal in 64.4% of the sample, OLD having predominated (in 32.2%). This suggested that obesity affected respiratory function. In addition, the proportion of normal spirometry results was lower in the individuals who were severely obese than in those who were overweight or obese.

Bronchodilator response was observed in 25.4% of the individuals studied, suggesting the presence of reversible OLD. Although 2 patients had a substantial bronchodilator response, neither had any disease to which airway hyperresponsiveness might have been attributed. The use of exhaled nitric oxide or induced sputum in order to assess airway inflammation might contribute to explaining this finding.

It has been reported that FEF_25-75%_ is a predictor of small airway obstruction.^(^ ^14^ ^)^ Pre- and post-bronchodilator FEF_25-75%_ were below the LLN in 51 (86.4%) and 41 (69.4%), respectively, of the individuals analyzed in the present study. This suggests that OLD and bronchodilator response occurred mainly at the level of the small airways. The diagnosis of OLD in the present study was primarily based on spirometric data rather than on TLC. This was due to the fact that only 2 of the participants had a TLC of more than 120% of predicted, a finding suggesting that the individuals with OLD had less hyperinflation and, therefore, less severe disease.

We found a significant negative correlation between the anthropometric and spirometric variables in the individuals with MORLD. However, this was found in only a small number of participants, further studies being therefore required.

We found studies describing and quantifying lung function changes, with conflicting results. Saxena et al.^(^ ^15^ ^)^ studied young adults and reported that lung function changes occur in overweight individuals and are proportional to the degree of obesity. According to Ora et al.,^(^ ^16^ ^)^ reduced lung volumes in adults appear to be associated with obesity and predispose to increased airway resistance and decreased expiratory flow.

Spathopoulos et al.^(^ ^17^ ^)^ in a cohort study involving children in Greece, investigated the influence of obesity on pulmonary function, as well as a possible association of obesity with atopy and asthma. Teixeira et al.^(^ ^18^ ^)^ found significant changes in the slow vital capacity and peak expiratory flow of obese children and adolescents. El-Baz et al.^(^ ^19^ ^)^ investigated the impact of obesity and body fat distribution on lung function in Egyptian children and found that respiratory symptoms were more common in the children who were overweight than in those who were normal weight. The authors also found significant RLD, small airway obstruction, respiratory muscle dysfunction, and increased airway resistance in the overweight children.^(^ ^19^ ^)^ However, no such association was found in other studies. Bertolini and Koseki^(^ ^20^ ^)^ found no correlation between lung function and anthropometric measurements in moderately obese children. Pekkarinen et al.^(^ ^21^ ^)^ investigated individuals over 18 years of age and found no correlation between body composition and spirometric changes; however, the authors reported a negative correlation between waist circumference and the FEV_1_/FVC ratio. Finally, according to Boran et al.,^(^ ^22^ ^)^ a likely explanation for these discrepant findings is the fact that most studies investigate extreme levels of obesity or have a small sample size, with no control groups.

The high prevalence of lung disease in the present study is consistent with some of the data from the studies cited herein, given that reduced chest compliance, increased abdominal pressure, early airway collapse, and increased airway resistance are expected in overweight individuals.^(^ ^23^ ^)^ These changes can explain why overweight individuals have lung disease (OLD, RLD, or MORLD). According to Lopes,^(^ ^24^ ^)^ inflammatory processes mediated by cytokines produced by adipocytes are responsible for pulmonary changes, characterized by airway hyperresponsiveness, among others. This observation is in agreement with those of El-Baz et al.,^(^ ^19^ ^)^ who defined fat as a metabolically active tissue.

In a study investigating biomarkers of lung function in overweight adolescents with asthma,^(^ ^25^ ^)^ weight loss was shown to reduce the concentration of adiponectin (an anti-inflammatory mediator), especially in cases of visceral obesity; to increase FEV_1_ and FVC; and to improve asthma symptoms. In a study investigating mildly obese children, Boran et al.^(^ ^22^ ^)^ reported that 3 of the children had reversible OLD (as determined by pulmonary function test results), despite having no previous respiratory symptoms or atopic diseases; the authors also reported that, given the lack of provocation tests, further studies were needed in order to determine whether obesity caused or increased airway hyperresponsiveness. In the present study, participants were not screened for atopy and no bronchial provocation tests were performed; however, the number of individuals with a bronchodilator response was higher in the present study than in the study conducted by Boran et al.^(^ ^22^ ^)^

The pathophysiology of obesity-related lung changes includes changes resulting from restricted lung expansion caused by lipid deposition, with decreased alveolar surface area, and reducing functional residual capacity.^(^ ^26^ ^)^ Therefore, the etiopathogenesis of OLD in obese individuals appears to involve mechanical and inflammatory processes. Studies have examined the association between asthma and obesity in an attempt to determine whether it is due to reduced lung volumes or increased airway resistance resulting in asthma-like symptoms.^(^ ^27^ ^)^

Camilo et al.^(^ ^28^ ^)^ reported that obesity is not the only factor responsible for the development and increased prevalence of asthma. Other important factors, such as genetic factors, immunological factors, and environmental factors, should be considered in future studies. Story^(^ ^29^ ^)^ reported that airway inflammation, mechanical changes secondary to obesity, and airway hyperresponsiveness, as well as changes in diet and physical activity, are related to the development of asthma in overweight individuals. Story^(^ ^29^ ^)^ also reported that obesity increases the severity of asthma and reduces the quality of life in obese children, having reported that further studies are needed in order to clarify the relationship between asthma and obesity. In our study, this finding was considered relevant, given that the study sample consisted of patients without respiratory disease or symptoms.

The present study has limitations that should be considered. One limitation is the number of participants, which can be partly explained by the difficulty in finding individuals meeting the inclusion criteria. Another limitation is related to the use of the helium dilution method for measuring lung volumes. The method is known to underestimate lung volumes by not measuring the volume of air in areas of air trapping excluded from ventilation. This, however, is of little or no significance, given that the study sample consisted of young patients with normal chest X-rays and no history of respiratory disease. Yet another limitation is the difficulty in choosing reference values for the age group under study. Because there are no universally recommended values, we used parameters that are generally accepted in the literature. Finally, there was no control group in the present study; Pereira^(^ ^12^ ^)^ established reference values, and those were used here. Therefore, lung function should be further investigated in longitudinal studies involving samples that are similar to our study sample, in order to determine the risk of lung disease by means of bronchial provocation tests, measurement of DLCO, and determination of immune, hormonal, and inflammatory mediators.

Because pulmonary function tests provide objective data on lung function,^(^ ^3^ ^)^ they are essential in the management of individuals with respiratory dysfunction and in individuals at risk of developing respiratory dysfunction, as is the case of overweight individuals. The combination of spirometry and lung volume measurements should be the method of choice for the evaluation of lung function, given that it is the best and most comprehensive method for measuring lung function, allowing a reliable diagnosis of lung disease. This combination should therefore be part of the routine care of such patients.

In conclusion, the prevalence of lung disease in overweight children and adolescents was high in the present study, OLD having predominated. The bronchodilator response observed in the present study was greater than was that reported in the literature, a positive bronchodilator response being more common in the individuals who were severely obese than in those who were obese or overweight.
