# Supplementary material for: Lung function in the absence of respiratory symptoms in overweight children and adolescents
Source: J Bras Pneumol. 2014 Mar-Apr;40(2):134–41. doi: 10.1590/S1806-37132014000200006 (PMC4083643; doi:10.1590/S1806-37132014000200006)
Supplement: Translation

# Translation: Função pulmonar de crianças e adolescentes sem sintomas respiratórios e com excesso de peso*

**Authors:** Silvana Neves Ferraz de Assunção, Carla Hilário da Cunha Daltro, Ney Christian Boa Sorte, Hugo da Costa Ribeiro, Maria de Lourdes Bastos, Cleriston Farias Queiroz, Antônio Carlos Moreira Lemos

**Affiliations:** Complexo Hospitalar Universitário Prof. Edgard Santos, Faculdade de Medicina da Bahia, Universidade Federal da Bahia, Salvador (BA) Brasil; Complexo Hospitalar Universitário Prof. Edgard Santos, Faculdade de Medicina da Bahia, Universidade Federal da Bahia, Salvador (BA) Brasil; Complexo Hospitalar Universitário Prof. Edgard Santos, Faculdade de Medicina da Bahia, Universidade Federal da Bahia, Salvador (BA) Brasil; Complexo Hospitalar Universitário Prof. Edgard Santos, Faculdade de Medicina da Bahia, Universidade Federal da Bahia, Salvador (BA) Brasil; Complexo Hospitalar Universitário Prof. Edgard Santos, Faculdade de Medicina da Bahia, Universidade Federal da Bahia, Salvador (BA) Brasil; Complexo Hospitalar Universitário Prof. Edgard Santos, Faculdade de Medicina da Bahia, Universidade Federal da Bahia, Salvador (BA) Brasil; Complexo Hospitalar Universitário Prof. Edgard Santos, Faculdade de Medicina da Bahia, Universidade Federal da Bahia, Salvador (BA) Brasil

**Keywords:** Obesidade/complicações, Testes de função respiratória, Pneumopatias/etiologia

## Abstract

**OBJETIVO:**

Descrever os achados de função pulmonar em crianças e adolescentes sem doenças respiratórias e com excesso de peso.

**MÉTODOS:**

Estudo transversal com crianças e adolescentes de 8 a 18 anos de ambos os sexos, com excesso de peso e sem doença respiratória, submetidos à avaliação antropométrica, radiografia de tórax, oximetria de pulso, espirometria e medidas de volume pulmonar. Indivíduos com patologias respiratórias, tabagistas ativos, radiografia anormal ou SpO_2_ = 92% foram excluídos do estudo. A circunferência da cintura foi medida em centímetros. O escore z para índice de massa corpórea/idade e sexo foi utilizado para classificar os indivíduos como com sobrepeso, obesos e obesos graves. As variáveis dos testes de função pulmonar foram expressas em percentual do previsto e correlacionadas com os índices antropométricos.

**RESULTADOS:**

Foram incluídos 59 indivíduos (30 meninos e 29 meninas). A média de idade foi de 11,7 ± 2,7 anos. Os resultados dos testes de função pulmonar foram normais em 21 indivíduos (35,6%). Dos 38 indivíduos restantes, 19 (32,2%), 15 (25,4%) e 4 (6,7%) apresentaram, respectivamente, distúrbio ventilatório obstrutivo, restritivo e misto. A resposta ao broncodilatador foi positiva em 15 indivíduos (25,4%), e a medida da CPT revelou que todos os indivíduos com CV reduzida apresentavam distúrbio ventilatório restritivo. Houve correlações negativas significantes entre os índices antropométricos e índice de Tiffeneau nos indivíduos com distúrbio ventilatório misto.

**CONCLUSÕES:**

A função pulmonar apresentou-se alterada em aproximadamente 65% dos indivíduos com sobrepeso aqui avaliados, predominando distúrbio ventilatório obstrutivo e resposta positiva ao broncodilatador.

## Introdução

A obesidade é uma doença crônica, de origem multifatorial e epidêmica no mundo.^(^ ^1^ ^)^ O interesse pelo seu conhecimento vem crescendo, tendo em vista o comprometimento social, econômico, assim como a morbidade e a mortalidade associada.^(^ ^2^ ^)^ Uma das possíveis complicações decorrentes da obesidade é a alteração da função pulmonar, o que muitas vezes não é percebido, embora ocorra de forma semelhante a outras doenças, como câncer, doenças cardiovasculares e doenças respiratórias crônicas.^(^ ^3^ ^)^ No estudo de Lin at al.,^(^ ^4^ ^)^ foi demonstrado que várias citocinas presentes na obesidade devem contribuir para a produção de efeitos inflamatórios sistêmicos em indivíduos com doença obstrutiva de vias aéreas e síndrome da apneia do sono.

A literatura tem descrito as alterações da função pulmonar consequentes à obesidade em adultos, mas esses dados são limitados na população infantil.^(^ ^5^ ^)^ O aumento da obesidade na infância tem se constituído um problema emergente no mundo contemporâneo e contribui de forma direta para sua manutenção na fase adulta; como consequência, há a elevação da incidência de doenças associadas à mortalidade, como as doenças cardiovasculares, a síndrome metabólica, a dislipidemia, o diabetes melito, a hipertensão arterial e, inclusive, as alterações respiratórias.^(^ ^6^ ^)^

Diferenças metodológicas referentes à avaliação diagnóstica da obesidade, bem como à seleção de uma população bem caracterizada, dificultam a comparação entre estudos, reforçando a necessidade de aprofundamento desse tema. Em virtude dessas considerações, o presente estudo teve como objetivo descrever as alterações funcionais pulmonares em crianças e adolescentes assintomáticos respiratórios com excesso de peso.

## Métodos

Estudo transversal e descritivo envolvendo pacientes com excesso de peso, com idade de 8-18 anos e de ambos os sexos. A idade foi computada em anos completos, e a cor foi classificada pelo pesquisador com base nos parâmetros do Instituto Brasileiro de Geografia e Estatística como negra, parda, branca, amarela e indígena.^(^ ^7^ ^)^ A amostra de conveniência foi obtida no período entre maio de 2010 e setembro de 2011, no ambulatório de obesidade infantojuvenil do Serviço de Nutrologia Pediátrica do Complexo Hospitalar Universitário Professor Edgard Santos, na cidade de Salvador (BA). Os participantes responderam a um questionário sistematizado criado para a pesquisa, no qual foram anotados dados da anamnese e do exame físico, com ênfase na avaliação do sistema respiratório e, em seguida, foram realizados os exames complementares. Assim, foram incluídos os indivíduos portadores de obesidade exógena, assintomáticos respiratórios, com ausculta pulmonar e radiografia de tórax normal e medida da SpO_2_ em repouso > 92%. Aqueles com relato de tabagismo ativo, assim como história de crises de sibilância, tosse, dor torácica ou doenças pulmonares conhecidas, não foram incluídos.

A circunferência da cintura foi medida com fita métrica maleável e inelástica, no ponto médio entre o último arco costal e a crista ilíaca anterossuperior.^(^ ^8^ ^)^ Foram realizadas duas medidas, e a média entre as duas foi adotada. O peso foi mensurado usando-se uma balança portátil (Filizola, São Paulo, Brasil), com sensibilidade de 100 g, com os participantes apenas com roupas íntimas. Um estadiômetro de base plana, com escala graduada em centímetros, com intervalos de 0,1 cm, foi usado para mensurar a altura. O cálculo do índice de massa corpórea (IMC) foi realizado pela fórmula peso/altura^2 ^(kg/m^2)^, e o escore z para IMC/idade e sexo da Organização Mundial da Saúde^(^ ^9^ ^)^ foi utilizado para classificar o estado antropométrico dos participantes nas categorias sobrepeso, obeso e obeso grave, caso apresentassem valores, respectivamente, acima de um, dois ou três desvios-padrão em relação à população de referência.

A avaliação da função pulmonar foi realizada no Laboratório de Função Pulmonar da Universidade Federal da Bahia através de espirometria com prova farmacológica, utilizando-se um espirômetro pneumotacógrafo Koko Digidoser (Ferraris Respiratory, Louisville, CO, EUA). As provas foram realizadas antes e após 15 min da inalação de salbutamol (400 µg). As medidas de VR e CPT foram realizadas pelo método de diluição do hélio, utilizando um sensor de fluxo de massa Vmax 21 (Viasys Healthcare, Palm Springs, CA, EUA). Os participantes foram orientados a evitar o consumo de café, chás, medicamentos (como broncodilatadores) e alimentos em grande quantidade antes dos exames, os quais foram realizados com os indivíduos com a cabeça em posição central e utilizando um clipe nasal. As provas ocorreram sempre no mesmo turno (vespertino) e foram realizadas pelo mesmo profissional. Foram feitas pelo menos três manobras de CVF para cada indivíduo, e foram aceitos os testes e curvas que apresentaram padrões aceitáveis e reprodutíveis segundo critérios da *American Thoracic Society**^(^* ^10^ ^)^ e da Sociedade Brasileira de Pneumologia e Tisiologia.^(^ ^11^ ^)^ Os valores espirométricos foram avaliados selecionando-se o maior de pelo menos três valores tecnicamente aceitos para o VEF_1_ e a CVF. Esses valores foram expressos em valores absolutos e percentuais sobre o predito, sendo esse definido como o limite inferior de normalidade (LIN) de cada indivíduo. Para o cálculo do LIN, foi utilizada a equação descrita por Pereira^(^ ^12^ ^)^ para ambos os sexos. Os valores de referência para os volumes pulmonares (VR e CPT) foram os descritos por Pennock et al.^(^ ^13^ ^)^

Os distúrbios ventilatórios foram definidos utilizando-se os valores espirométricos, a CPT e o VR, como segue:

- Espirometria normal: CVF = LIN; VEF_1_/CVF = LIN; VR e CPT normais
- Distúrbio ventilatório obstrutivo (DVO): VEF_1_/CVF < LIN; VR ou CPT normal ou aumentada; ou resposta positiva ao broncodilatador
- Distúrbio ventilatório restritivo (DVR): CVF < LIN; VEF_1_/CVF = LIN; VR e CPT reduzidos
- Distúrbio ventilatório misto (DVM): VEF_1_/CVF < LIN e CPT reduzida

O teste de resposta ao broncodilatador seguiu as diretrizes da Sociedade Brasileira de Pneumologia e Tisiologia,^(^ ^11^ ^)^ e a resposta foi considerada significativa quando:

- Resultados alterados da espirometria apresentassem uma diferença, em valor absoluto, do VEF_1_ após o uso de broncodilatador (pós-Bd) - VEF_1_ antes do uso de broncodilatador (pré-Bd) = 200 mL e o percentual de aumento do valor previsto [(VEF_1_ pós-Bd - VEF_1_ pré-Bd) × 100/VEF_1_, % previsto] > 7%. Em relação à CVF, a diferença entre os valores pós-Bd e pré-Bd, em valor absoluto, resultasse = 350 mL.
- Resultados normais da espirometria apresentassem uma variação do VEF_1_ pós-Bd - VEF_1_ pré-Bd = 10%.

Para a análise dos dados, utilizou-se o pacote estatístico IBM SPSS Statistics, versão 19.0 (IBM Corporation, Armonk, NY, EUA). As variáveis quantitativas foram expressas em média e desvio-padrão. As variáveis qualitativas foram expressas em frequência simples e relativa. O teste de Pearson foi indicado para o estudo da correlação entre as variáveis espirométricas, escore z para IMC/idade e circunferência da cintura para cada grupo de distúrbio ventilatório (obstrutivo, restritivo e misto). Foram considerados significantes valores de p = 0,05. O estudo foi aprovado pelo comitê de ética da instituição (parecer 80/09) e o termo de consentimento livre e esclarecido foi obtido de todos os participantes por meio dos seus representantes legais.

## Resultados

A amostra foi composta por 59 indivíduos, com média de idade de 11,7 ± 2,7 anos. Desses, 30 (50,8%) eram do sexo masculino. Quanto à cor, 32 indivíduos (54,2%) apresentaram cor parda, 22 (37,2%) eram negros, e 5 (8,4%) eram brancos. O escore z para IMC/idade variou de 1,2 a 6,1 dp, com média de 3,1 ± 1,0 (kg/m^2)^, resultando na classificação de sobrepeso, em 4 indivíduos (6,7%); obesidade, em 28 (47,4 %); e obesidade grave, em 27 (45,7%). Os resultados das medidas de função pulmonar do estudo, em % do previsto, estão detalhados na Tabela 1.

**Tabela 1:** Medidas de função pulmonar, em percentual do previsto, de 59 crianças e adolescentes com excesso de peso categorizados pelo tipo de distúrbio ventilatório.a

Distúrbio ventilatório	VEF_1_		CVF		VEF_1_/CVF		FEF_25-75%_		CPT	VR
	Pré-Bd	Pós-Bd	Pré-Bd	Pós-Bd	Pré-Bd	Pós-Bd	Pré-Bd	Pós-Bd	Pré-Bd	Pré-Bd
Normal (sem distúrbio ventilatório)	95,43 ± 10,89	95,10 ± 12,19	93,05 ± 9,51	90,16 ± 11,40	86,81 ± 3,44	88,67 ± 2,79	64,86 ± 14,38	66,86 ± 15,14	91,40 ± 8,70	89,65 ± 27,43
Obstrutivo	86,26 ± 13,23	89,74 ± 17,19	93,06 ± 13,51	92,95 ± 15,50	76,42 ± 5,09	80,47 ± 7,40	51,42 ± 14,35	60,63 ± 22,30	99,00 ± 13,55	118,56 ± 47,53
Restritivo	75,33 ± 13,08	79,27 ± 14,54	72,20 ± 14,30	74,27 ± 15,56	88,80 ± 4,88	89,21 ± 5,80	57,33 ± 12,88	64,20 ± 18,27	69,38 ± 7,95	66,62 ± 42,53
Misto	73,50 ± 5,91	80,50 ± 12,15	83,25 ± 4,19	86,00 ± 13,24	74,25 ± 3,86	78,75 ± 3,86	45,25 ± 8,57	55,50 ± 14,40	71,75 ± 11,89	37,25 ± 41,99

Bd : broncodilatador

a Valores expressos em média ± dp.

Observamos que 17 indivíduos (30,3%) apresentaram valores de CPT < 80% do previsto, enquanto 2 (3,5%) apresentaram CPT > 120%.

Analisando a resposta ao broncodilatador, observamos que 15 indivíduos (25,4%), sendo 10 (67,0%) obesos graves, apresentaram resposta positiva verificada pela variação do VEF_1 _(Tabela 2). Todos eles apresentaram valores da relação VEF_1_/CVF abaixo do LIN, portanto, com DVO. Em relação à variação pré-Bd e pós-Bd da CVF, apenas 2 indivíduos apresentaram resposta positiva, e nenhum paciente com DVM apresentou resposta ao broncodilatador. Um dos indivíduos com resposta positiva não conseguiu realizar a medida dos volumes pulmonares.

**Tabela 2:** Valores das variáveis de função respiratória, em percentual do previsto, de 15 crianças e adolescentes que apresentaram variação do VEF1.

Sujeitos	VEF_1_		CVF		VEF_1_/CVF		FEF_25-75%_		CPT	VR
	Pré-Bd	Pós-Bd	Pré-Bd	Pós-Bd	Pré-Bd	Pós-Bd	Pré-Bd	Pós-Bd	Pré-Bd	Pré-Bd
01	85	94	101	102	71	78	53	65	95	62
02	84	91	87	89	79	84	58	75	86	78
03	94	102	100	101	78	83	72	80	94	71
04	101	110	108	112	80	83	70	81	104	99
05	103	110	111	114	78	82	69	82	119	150
06	100	108	101	100	81	89	80	110	89	31
07	70	94	70	95	85	83	33	40		
08	69	78	83	91	70	73	35	43	79	70
09	68	78	75	78	75	80	38	47	84	106
10	92	103	112	108	69	79	48	60	124	171
11	84	96	83	90	84	88	69	69	80	74
12	80	98	89	102	77	81	56	74	78	46
13	86	101	94	92	78	93	51	75	128	235
14	86	97	87	93	82	86	62	78	104	169
15	70	82	68	70	95	100	44	80	73	103

Bd : broncodilatador.

Considerando o VEF_1 _como o parâmetro preditor de obstrução brônquica, a sua variação em valores absolutos e relativos após o uso do broncodilatador foi analisada e encontra-se detalhada na Tabela 3.

**Tabela 3:** Análise da variação do VEF1 nas 15 crianças e adolescentes com excesso de peso que apresentaram resposta ao broncodilatador.

Sujeitos	ΔVEF_1_, mL*	ΔVEF_1_, %**
01	260	9
02	180	7
03	310	8
04	260	9
05	210	7
06	350	8
07	420	24
08	270	9
09	240	7
10	290	11
11	310	12
12	570	18
13	380	15
14	370	11
15	260	12

* Média ± dp = 312 ± 96,7 mL

** Média ± dp = 11,1 ± 4,7%.

Tendo em vista a predominância de distúrbios ventilatórios na amostra estudada, verificou-se a sua ocorrência nos diferentes graus de obesidade, como se observa na Tabela 4.

**Tabela 4:** Distribuição do tipo de distúrbio ventilatório de acordo com os graus de obesidade.a

Tipos	Sobrepeso/obeso	Obesos graves	Total
Normal	14 (23,7)	07 (11,8)	21 (35,5)
DVO	09 (15,2)	10 (16,9)	19 (32,2)
DVR	07 (11,8)	08 (13,5)	15 (25,4)
DVM	02 (3,3)	02 (3,3)	04 (6,7)

DVO : distúrbio ventilatório obstrutivo

DVR : distúrbio ventilatório restritivo

DVM : distúrbio ventilatório misto

a Valores expressos em n (%).

A análise de correlação de Pearson*,* realizada entre o IMC, a circunferência da cintura e as variáveis espirométricas em relação aos distúrbios ventilatórios, é mostrada na Tabela 5. Observa-se uma correlação negativa estatisticamente significativa entre esses parâmetros antropométricos e a relação VEF_1_/CVF nos indivíduos com DVM.

**Tabela 5:** Análise das correlações entre as variáveis antropométricas e espirométricas em relação aos diferentes distúrbios ventilatórios.

Variáveis espirométricas	Variáveis antropométricas												
	Circunferência da cintura							Índice de massa corpórea					
	DVO		DVR		DVM			DVO		DVR		DVM	
	r	p	r	p	r	p		r	p	r	p	r	p
VEF_1_	0,064	0,795	0,331	0,228	0,628	0,371		-0,092	0,709	0,146	0,603	0,434	0,566
CVF	0,048	0,845	0,250	0,369	0,645	0,355		-0,127	0,603	0,091	0,747	0,461	0,539
VEF_1_/CVF	0,095	0,700	0,113	0,689	-0,996	0,004		-0,219	0,367	0,106	0,708	-0,986	0,014
FEF_25-75%_	0,055	0,823	0,265	0,341	-0,667	0,333		0,203	0,405	0,358	0,190	-0,527	0,473

DVO : distúrbio ventilatório obstrutivo

DVR : distúrbio ventilatório restritivo

DVM : distúrbio ventilatório misto

## Discussão

No presente estudo, foi avaliada a função pulmonar de uma população jovem, com excesso de peso, assintomáticos respiratórios, sem doença pulmonar prévia e com radiografia de tórax normal. Os testes de função pulmonar mostraram-se alterados em 64,4% da amostra, com predomínio de DVO (32,2%), sugerindo que a obesidade influenciou a função ventilatória. Adicionalmente, os autores, quando compararam esses achados de função pulmonar com os graus de obesidade, verificaram uma menor proporção de espirometrias normais em obesos graves em relação àqueles classificados como com sobrepeso ou obesos.

A resposta ao broncodilatador foi verificada em 25,4% dos indivíduos, sugerindo a presença de DVO com reversibilidade. Em relação ao fato de 2 pacientes apresentarem uma resposta acentuada ao broncodilatador, não foi encontrada, na avaliação desses pacientes, nenhuma patologia que justificasse hiper-responsividade de vias aéreas. É possível que a avaliação da inflamação das vias aéreas pelo óxido nítrico exalado ou pelo escarro induzido possa contribuir para a elucidação desse achado.

A observação detalhada dos dados espirométricos dos indivíduos estudados revelou que o FEF_25-75%_, um preditor de obstrução das vias aéreas de pequeno calibre,^(^ ^14^ ^)^ antes e após o uso do broncodilatador, respectivamente, apareceu abaixo do LIN em 51 (86,4%) e em 41 (69,4%) dos indivíduos analisados. Esse dado sugere que o DVO e a resposta ao broncodilatador ocorreram principalmente no nível das pequenas vias aéreas. Salientamos ainda que o DVO foi diagnosticado levando-se mais em conta os dados espirométricos do que pela CPT, já que apenas 2 participantes apresentaram CPT > 120%, sugerindo que os indivíduos com DVO mostraram menor hiperinsuflação e, portanto, menor gravidade no presente estudo.

Foi também demonstrada uma correlação negativa significante entre as variáveis antropométricas e espirométricas envolvendo indivíduos com DVM. Ressaltamos que esse achado ocorreu em pequeno número de participantes, havendo, portanto, a necessidade de novas investigações nesse sentido.

Na literatura aqui utilizada, encontramos pesquisas que relatam as disfunções sobre o sistema respiratório com o intuito de descrever e quantificar essas alterações, com resultados diferentes e conflitantes. Saxena et al.,^(^ ^15^ ^)^ estudando adultos jovens, afirmam que repercussões funcionais respiratórias ocorrem em indivíduos com excesso de peso e que essas são proporcionais ao grau de obesidade. Para Ora et al.,^(^ ^16^ ^)^ a redução dos volumes pulmonares em adultos parece estar associada à obesidade e predispõe a um aumento da resistência das vias aéreas e redução do fluxo expiratório.

Spathopoulos et al.,^(^ ^17^ ^)^ em uma coorte envolvendo crianças na Grécia, investigaram a influência da obesidade na função pulmonar, bem como uma possível ligação entre obesidade, atopia e asma. Adicionalmente, Teixeira et al.,^(^ ^18^ ^)^ estudando crianças e adolescentes, encontraram alterações significativas na capacidade vital lenta e no pico de fluxo expiratório e relacionaram esses achados à obesidade. El-Baz et al.,^(^ ^19^ ^)^ investigando o impacto da obesidade na função pulmonar, bem como sua correlação com o grau e a distribuição da obesidade em crianças no Egito, encontraram sintomas respiratórios mais frequentes, DVR significante, obstrução de pequenas vias aéreas, defeito na musculatura respiratória e aumento na resistência das vias aéreas nas crianças com excesso de peso quando comparadas com aquelas com peso normal. Por outro lado, outros estudos não demonstram essa associação. Bertolini e Koseki^(^ ^20^ ^)^ verificaram que não houve correlações entre a função pulmonar e achados antropométricos na obesidade infantil moderada. Pekkarinen et al.,^(^ ^21^ ^)^ estudando indivíduos maiores de 18 anos, não relacionaram a composição corporal às alterações espirométricas, embora retenham relatado uma correlação negativa entre a circunferência da cintura e a relação VEF_1_/CVF. Finalmente, Boran et al.^(^ ^22^ ^)^ argumentam que uma provável explicação para esses achados discrepantes pode ser o fato de que a maioria dos estudos diz respeito a níveis extremos de obesidade ou possui um tamanho amostral pequeno, sem grupos controle.

A alta prevalência de distúrbios ventilatórios observada no presente estudo é concordante com alguns dados da literatura citada, uma vez que em indivíduos com excesso de peso é esperado uma redução da complacência torácica, aumento da pressão abdominal, colapso mais precoce das vias aéreas e aumento da resistência das vias aéreas.^(^ ^23^ ^)^ Tais alterações podem explicar o fato de indivíduos com excesso de peso apresentar algum tipo de distúrbio ventilatório, seja DVO, DVR ou DVM. Segundo Lopes,^(^ ^24^ ^)^ processos inflamatórios mediados por citocinas produzidas pelos adipócitos responderiam pelos quadros de alterações pulmonares, caracterizados por hiper-responsividade de vias aéreas, entre outros. Essa observação é compartilhada por El-Baz et al.,^(^ ^19^ ^)^ que definem a gordura como um tecido metabolicamente ativo.

Em um estudo envolvendo a investigação de biomarcadores na função pulmonar de adolescentes asmáticos com excesso de peso,^(^ ^25^ ^)^ houve uma redução do mediador anti-inflamatório adiponectina, principalmente na obesidade visceral, e um aumento de VEF_1_ e CVF, bem como melhora da asma, com a perda de peso dos adolescentes. Boran et al.^(^ ^22^ ^)^ argumentam, em seu estudo com crianças, que embora 3 dos pacientes tenham apresentado alteração obstrutiva reversível em seus testes de função pulmonar, eles não tinham apresentado sintomas respiratórios prévios ou atopias, e, uma vez que não foi realizado nenhum teste de provocação, havia a necessidade de mais estudos a fim de se determinar se a obesidade causaria ou acentuaria a hiper-responsividade das vias aéreas. No presente estudo, não foram realizados a avaliação de atopia ou o teste de broncoprovocação; no entanto, houve um maior número de indivíduos responsivos ao broncodilatador que no estudo de Boran et al.^(^ ^22^ ^)^

A fisiopatologia da obesidade relacionada ao sistema ventilatório descreve alterações resultantes da restrição à expansibilidade pulmonar pela deposição lipídica, com diminuição da superfície alveolar, comprometendo a capacidade residual funcional.^(^ ^26^ ^)^ Assim, a etiopatogenia dos DVOs presentes na obesidade parece envolver processos mecânicos e inflamatórios. Debates sobre a associação entre asma e obesidade procuram explicar se esse achado ocorre pela redução dos volumes pulmonares ou pelo aumento da resistência das vias aéreas, resultando em sintomas semelhantes à asma.^(^ ^27^ ^)^

Camilo et al.^(^ ^28^ ^)^ chamam a atenção para o fato de que a obesidade, por si só, não constitui a única etiologia responsável pelo desenvolvimento e pelo aumento na prevalência da asma. Outros fatores importantes, como os genéticos, imunológicos e ambientais, devem ser considerados em estudos. Story^(^ ^29^ ^)^ descreveu que a inflamação das vias aéreas, as alterações mecânicas secundárias à obesidade e a hiper-responsividade das vias aéreas, bem como mudanças nas atividades físicas e na dieta, estariam relacionados ao aparecimento da asma em indivíduos com excesso de peso. Story^(^ ^29^ ^)^ relatou também que a obesidade aumenta a gravidade da asma e reduz a qualidade de vida da criança portadora e que são necessários mais estudos para definir claramente a relação entre asma e obesidade. Em nosso estudo, esse achado foi considerado relevante, uma vez que a amostra selecionada foi constituída por pacientes sem doença pulmonar ou sintomas respiratórios.

Prováveis limitações no presente estudo devem ser consideradas. Uma delas refere-se ao número de participantes, em parte explicado pela dificuldade de seleção de indivíduos que preenchessem os critérios de inclusão. Outro aspecto relevante diz respeito ao método de diluição do hélio utilizado para medida dos volumes pulmonares, reconhecido por não medir o volume de ar nas áreas com aprisionamento aéreo excluídas da ventilação, subestimando, assim, essas medidas. No entanto, por tratar-se de pacientes jovens, sem doença pulmonar prévia e com radiografia de tórax normal, esse fato é inexpressivo ou minimizado. Citamos também a dificuldade que envolve a escolha de valores de referência para a faixa etária envolvida na pesquisa, uma vez que não existem valores universalmente preconizados, o que foi contornado pela utilização de parâmetros aceitos na literatura. Finalmente, não foi constituído um grupo controle, por considerarmos que já existem valores preditos para a normalidade recomendados por Pereira,^(^ ^12^ ^)^ aqui utilizados. Assim, recomenda-se prosseguir na investigação da função pulmonar através de estudos longitudinais, com amostras semelhantes à estudada, a fim de verificar o seu risco futuro de desenvolver doenças pulmonares através de testes de broncoprovocação, medida da DLCO e dosagem de mediadores imunológicos, hormonais e inflamatórios.

Os testes de função pulmonar revelam-se fundamentais no manejo de indivíduos com disfunção respiratória, como também naqueles com risco potencial de desenvolvê-las, como os portadores de excesso de peso, pois fornecem dados objetivos sobre a função pulmonar.^(^ ^3^ ^)^ A associação da espirometria com a medida dos volumes pulmonares deve ser o método escolhido para a avaliação proposta, por essa ser o melhor e mais completo instrumento para aferir a função pulmonar, permitindo um fidedigno diagnóstico do distúrbio ventilatório. Tal associação deve, portanto, fazer parte da rotina assistencial desses pacientes.

Em conclusão, observou-se que, no presente estudo, indivíduos assintomáticos respiratórios com excesso de peso apresentaram uma alta prevalência de distúrbios ventilatórios, predominantemente DVO. Ademais, houve resposta positiva ao broncodilatador superior ao relatado na literatura e tal resposta ocorreu em maior frequência nos obesos graves.
